# Suppression of nicotinamide phosphoribosyltransferase expression by miR-154 reduces the viability of breast cancer cells and increases their susceptibility to doxorubicin

**DOI:** 10.1186/s12885-019-6221-0

**Published:** 2019-11-01

**Authors:** Zahra Bolandghamat Pour, Mitra Nourbakhsh, Kazem Mousavizadeh, Zahra Madjd, Seyedeh Sara Ghorbanhosseini, Zohreh Abdolvahabi, Zahra Hesari, Samira Ezzati Mobasser

**Affiliations:** 1 Department of Molecular Medicine, Faculty of Advanced Technologies in Medicine, Iran University of Medical Sciences, Hemmat Highway 1449614535, Tehran, Iran; 2 Department of Biochemistry, Faculty of Medicine, Iran University of Medical Sciences, Tehran, Iran; 3 Cellular and Molecular Research Center, Faculty of Medicine, Iran University of Medical Sciences, Tehran, Iran; 4 Oncopathology Research Center, Iran University of Medical Sciences, Tehran, Iran; 5 Department of Biochemistry, Faculty of Pharmacy, Isfahan University of Medical Sciences, Isfahan, Iran; 6 Department of Biochemistry and Genetics, Cellular and Molecular Research Center, Qazvin University of Medical Sciences, Qazvin, Iran; 7 Laboratory Sciences Research Center, Golestan University of Medical Sciences, Gorgan, Iran; 8 Department of Laboratory Sciences, Faculty of Paramedicine, Golestan University of Medical Sciences, Gorgan, Iran

**Keywords:** Nicotinamide phosphoribosyltransferase, Breast cancer, miR-154, Doxorubicin, microRNA

## Abstract

**Background:**

Nicotinamide phosphoribosyltransferase (NAMPT) enzyme acts as the major enzyme in the nicotinamide adenine dinucleotide (NAD) synthesis salvage pathway. Deregulation of NAD could be associated with progression of several cancers such as breast cancer. Here, the consequence of NAMPT inhibition by miR-154 was investigated on breast cancer cells.

**Methods:**

MDA-MB-231 and MCF-7 cancer cell lines were transfected with the mimic and inhibitors of miR-154-5p and their corresponding negative controls. Consequently, levels of NAMPT and NAD were assayed employing qRT-PCR, Western blotting and enzymatic method, respectively. Subsequently, flow cytometry and colorimetric methods were performed to evaluate apoptosis and cell viability. Bioinformatics analyses as well as luciferase assay were done to investigate whether the 3′-UTR of NAMPT is directly targeted by miR-154.

**Results:**

According to the obtained results, NAMPT was recognized as a target for binding of miR-154 and the levels of this miRNA was inversely associated with both mRNA and protein levels of NAMPT in breast cancer cell lines. Functionally, miR-154 inhibited the NAD salvage pathway leading to a remarkable decrease in cell viability and increased rate of cell death. When breast cancer cells were simultaneously treated with doxorubicin and miR-154 mimic, cell viability was considerably reduced compared to treatment with doxorubicin alone in both cell lines.

**Conclusions:**

It was concluded that the inhibition of NAD production by miR-154 might be introduced as an appropriate therapeutic approach in order to improve breast cancer outcome either alone or in combination with other conventional chemotherapeutic agents.

## Background

Breast cancer is known as the most frequently diagnosed and the leading cause of cancer mortality in women globally [1, 2]. In spite of substantial progresses in breast cancer treatment, detection of novel therapeutic targets for overcoming current obstacles is still required. In recent years, inhibition of cellular and molecular mechanisms that interfere with development of breast cancer is one of the critical diagnostic and therapeutic strategies [1–3]. Nicotinamide phosphoribosyltransferase (NAMPT) is known as the rate-limiting enzyme in the salvage biosynthetic pathway of nicotinamide adenine dinucleotide (NAD) [4, 5] and is considered a critical enzyme that plays important roles in an extensive range of biological activities such as metabolism and immune response. Deregulation of NAMPT expression is related to initiation and progression of various human malignancies [6]. NAMPT provides NAD as the substrate for sirtuin enzymes that are upstream regulators of the expression of numerous genes by their deacetylase activity [7]. On the other hand, NAMPT increases the activity of estrogen receptor and thus facilitates breast cancer propagation [8]. Some studies have indicated that inhibition of NAMPT expression is associated with a remarkable increase in metabolic collapse and apoptosis in breast cancer cell lines both in vivo and in vitro [9]. On the contrary, up-regulation of NAMPT in breast cancer patients is closely related to poor response to chemotherapeutic drugs such as doxorubicin [10]. Hence, it seems that inhibition of NAMPT could provide cutting-edge therapeutic strategies for breast cancer treatment.

Among various cellular and molecular targets involved in breast cancer pathogenesis, microRNAs (miRNAs) are proved to act as key epigenetic regulators [11]. These molecules are known as a class of short non-coding RNAs that have effective roles in the adjustment of various biological functions including growth, angiogenesis, development, and differentiation [12]. Studies have indicated that deregulation of miRNAs is directly related to the emergence of various aspects of tumorigenesis such as angiogenesis, tumor growth, metastasis, and response to therapy in breast cancer [13, 14]. miR-154 is a tumor suppressor which is located at chromosome 14q32 [14]. Down-regulation of miR-154 is associated with progression of many cancers such as cancers of breast [14], prostate [15], osteosarcoma [16], hepatocellular carcinoma [17], thyroid [18] colorectal [19], and non-small cell lung cancer [20]. However, the function and underlying cellular and molecular pathways related to miR-154 has not been determined in breast cancer. In this research, we accomplished bioinformatics analysis and found that in the 3′-UTR of NAMPT mRNA, there is a binding site for miR-154. Then, we investigated if the enhancement of miR-154 could reduce NAD levels and suppress breast cancer cell propagation via targeting NAMPT and whether this inhibition could modulate the cellular response to doxorubicin (DOX).

## Methods

### Cell lines and cell culture

Four cell lines including MCF-7, MCF-10A, MDA-MB-231 and HEK-293 T were collected in 2017 from the Cell Bank of the Iranian Biological Resource Center (Tehran, Iran). The cell lines were authenticated using STR profiling analysis (STR identifiler PCR kit, Thermofisher, USA) and also evaluated for mycoplasma contamination by Hoechst staining as well as PCR. Viral and other bacterial infections were also assessed and ruled out. Dulbecco’s Modified Eagle’s Medium (DMEM, Biosera, France) was used for culturing MDA-MB-231 and MCF-7 cells. Mammary epithelial cell growth medium (MEGM;Lonza/ Clonetics, Switzerland) and DMEM/F12 (Biosera, France) were employed for culturing MCF-10A and HEK-293 T cells, respectively. Penicillin-streptomycin (1%) and fetal bovine serum (FBS) (10%) (Invitrogen, UK) were also included in cell culture media. For culturing of MCF-10A cells, other supplements including insulin (10 μg/ml), hydrocortisone (0.5 μg/ml), epithelial growth factor (20 ng/ml) and cholera toxin (100 ng/ml) (all from Sigma-Aldrich, Germany) were added up to MEGM. Finally, all cell lines were maintained at 37 °C in an incubator that was humidified with water and contained 5% CO_2_.

### Cell transfection

In order to perform cell transfection, polyethylenimine (PEI) (Sigma-Aldrich, Germany) was employed. To increase and decrease the level of miR-154-5p, we used microRNA mimic and microRNA inhibitor, respectively. These microRNAs were acquired from GenePharma (Shanghai, China). The sequences of miRNA mimic and inhibitor as well as their negative controls (i.e. NC inhibitor and NC mimic) are shown in Table 1. Cells were seeded into 6-, 12- or 96-well plates and incubated as previously described. Then, fresh FBS- and antibiotic-free medium were added and the plates were incubated for another 4 h. The PEI and oligonucleotides were mixed in Opti-MEM (Gibco, UK) and incubated for 40 min at 25 °C and this mixture was subsequently added to each well followed by incubation of the plate at 37 °C with 5% CO_2_ for 4 h. In order to evaluate transfection, fluorescein-conjugated miRNAs were used and the transfected cells were observed under a fluorescence microscope (Olympus, Japan) 8–24 h after transfection. ImageJ software (ImageJ, NIH, USA) was used for image analysis.

**Table 1:** Sequences of miR-154 mimic, miR-154 inhibitor and their negative controls

MicroRNA	Seq. (5′-3′)
microRNA Inhibitor N.C	5′-CAGUACUUUUGUGUAGUACAA-3′
microRNA mimic N.C	5′-UUGUACUACACAAAAGUACUG-3′
miR-154 Inhibitor	5′-CGAAGGCAACACGGAUAACCUA-3
miR-154 mimic	5′-UAGGUUAUCCGUGUUGCCUUCG-3

### RNA isolation and real-time RT-PCR

For evaluation of gene expression at the mRNA level, real-time-PCR was employed. miRCURY™ RNA isolation kit (Exiqon, USA) was used to extract total RNA from different cells. Afterwards, 1 μg RNA was used as template to synthesize complementary DNA (cDNA). Prior to cDNA synthesis, both the quantity and quality of the extracted RNA was spectrophotometerically analyzed (Nanodrop, Thermo Fisher Scientific, USA). In order to synthesize miR-154 cDNA, at first step, the 3′-end of the microRNAs was polyadenylated by Poly A Polymerase (PAP) from E.coli (New England Biolabs, UK). A hybrid primer having an adapter sequence and complementary sequence for the poly A tail was used for cDNA synthesis. The SYBR Green kit (SYBR Premix Ex Taq II, Takara, Japan) was used for performing the PCR. Glyceraldehyde 3-phosphate dehydrogenase (GAPDH) and human U6 small nuclear RNA gene expression levels were also assessed and used for normalizing the expression of NAMPT and miR-154, respectively. Each sample was analyzed in triplicate. Determination of the gene expression levels relative to the controls was calculated using the 2^-ΔΔCT^ formula. Additional file 1: Table S1 lists the used primer sequences.

### Cell survival assay

To evaluate the influence of miR-154 mimic and its inhibitor on cell survival, viability assay was performed employing tetrazolium based WST-1 reagent (Roche Applied Science, Germany). A cell suspension containing total number of 5 × 10^3^ cells/100 μl was seeded into every well of a 96-well plate. After overnight incubation of cells, they were transfected using either mimic or inhibitor of miR-154. Relevant negative controls were also included in transfection experiments. Finally, WST-1 reagent (10 μl/well) was added and after 4 h, optical density was measured by a plate reader (BioTek Instruments Inc., Winooski, USA) at 450/650 nm wavelength. For assessment of the effect of doxorubicin on cell viability, first a 2 mg/ml stock solution of doxorubicin (Cell signaling technology, USA) in water was prepared. Subsequently, either un-transfected cells or cells transfected with different miR-154-related oligonucleotides were treated with 0.1 μM concentration of doxorubicin for 24 h and the cell survival was evaluated as described above.

### Apoptosis assay

The effect of miR-154 on cell apoptosis was assessed by employing a flow-cytometric detection kit containing FITC-Annexin V and propidium iodide (PI) (Roche Applied Science, Germany) following the instructions provided by the kit. A total of 3 × 10^5^ of MCF-7 and MDA-MB-231 cells were seeded in 6-well plates and after 48 h, cells were harvested and after washing twice with PBS, were stained with Annexin V as well as PI. Finally, evaluation of stained cells was done by flow-cytometry (FACScan, BD Biosciences, USA) equipped with bandpass filters at 515 nm for detection of FITC, 600 nm for PI detection and 488 nm laser for excitation.

CellQuest software (BD Biosciences) was employed for estimation of obtained results. The cells that were positive for Annexin V-FITC were presented as cells that had undergone apoptosis.

### Western blotting

Western blot technique was used to investigate the effect of miR-154 alterations on NAMPT expression levels in the breast cancer cell lines. After 48 h, RIPA lysis buffer containing protease inhibitor (0.1%), phosphatase inhibitor (0.5%) and phenylmethanesulfonyl fluoride (PMSF) (10%) (all from Sigma-Aldrich, Germany) was used for cell lysis. Then, lysates (40 μg of the total protein from each sample) was subjected to sodium dodecylsulfate polyacrylamide gel electrophoresis (SDS-PAGE) on polyacrylamide gel (10%). In the next step, separated proteins were trans-blotted onto polyvinylidene difluoride (PVDF) membrane. After blocking of membranes in blocking buffer containing 0.05% Tween 20 and 5% powdered non-fat milk in PBS, the membranes were incubated with a PBEF/NAMPT Rabbit antibody at 1:1000 concentration (cell signaling technology, USA) followed by incubation with HRP-conjugated secondary antibody against rabbit IgG, at a dilution of 1:5000 (Cell Signaling Technology, USA). NAMPT protein and GAPDH (control) bands were detected by enhanced chemiluminescent reagent (Amersham Biosciences/GE Healthcare, U.K). Analysis of the resulting bands was performed by densitometry using ImageJ software (v1.52, NIH). For this purpose, the first band was primarily selected by drawing a rectangular shape around it and assigning it as the first lane. Then it was proceeded by selection of the other bands by moving the same rectangle to make sure that all selections have the same dimensions. Subsequently, the lane profile plot was generated. The area under the curve of each plot was determined which corresponded to the density of each band.

### NAD level assay

The concentration of intracellular NAD/NADH was measured using NAD assay kit (Abcam, UK) according to the instructions provided by the manufacturer. After lysis of the transfected cells using lysis buffer, the concentration of the total protein in the lysate was determined using a bicinchoninic acid (BCA) protein assay kit (Thermo Fisher Scientific, USA). Subsequently, the proteins were removed using perchloric acid and the NAD levels were obtained after enzymatic reaction, by measuring the absorbance at 450 nm. The quantity of NAD was normalized against the protein content in each sample.

### Prediction of candidate miRNAs targeting NAMPT 3′-UTR

For prediction of miRNAs that potentially bind to NAMPT 3′-UTR, the universally cited prediction programs were used including microRNA.org (miRanda algorithm) (www.microRNA.org), TargetScan (http://targetscan.org), and MiRmap (http://mirmap.ezlab.org). The score of binding affinities were obtained and compared with the other miRNAs.

### Investigation of miR-154-target interaction

To check the direct interaction of miR-154 with NAMPT 3′-UTR, the sequence of the 3'-UTR including the target binding site was cloned in the psiCHECK-2 reporter plasmid as previously described [21]. Briefly, the NAMPT 3′-UTR sequence was first determined using Gene database of PubMed (https://www.ncbi.nlm.nih.gov/gene). The relevant region was amplified by the primers listed in Additional file 1: Table S1; synthesized by Macrogen Inc., South Korea. The tandem mutant of NAMPT 3′-UTR was also constructed to serve as a negative control. To create this sequence, the forward and reverse primers containing restriction sites for NotI and XhoI were used (Additional file 1: Table S1). To ensure the presence of fused sequences and the absence of mutations in the sequence, the recombinant plasmid was sequenced by Macrogen Inc. The recombinant constructs were co-transfected with different miR-154-related oligonucleotides into HEK293T cell line. The activity of Renilla luciferase was measured over the activity of firefly by dual luciferase assay kit obtained from Promega (USA).

### Statistical analysis

All statistical analyses were done using GraphPad Prism software, version 5.01 (USA, San Diego). The differences between the experimental groups were evaluated by one way- ANOVA. All results were presented as mean ± standard deviation (S.D.). *P* values lower than 0.05 were recognized statistically significant.

## Results

### The expression levels of miR-154 and NAMPT in breast cancer cell lines

**Fig. 1:**
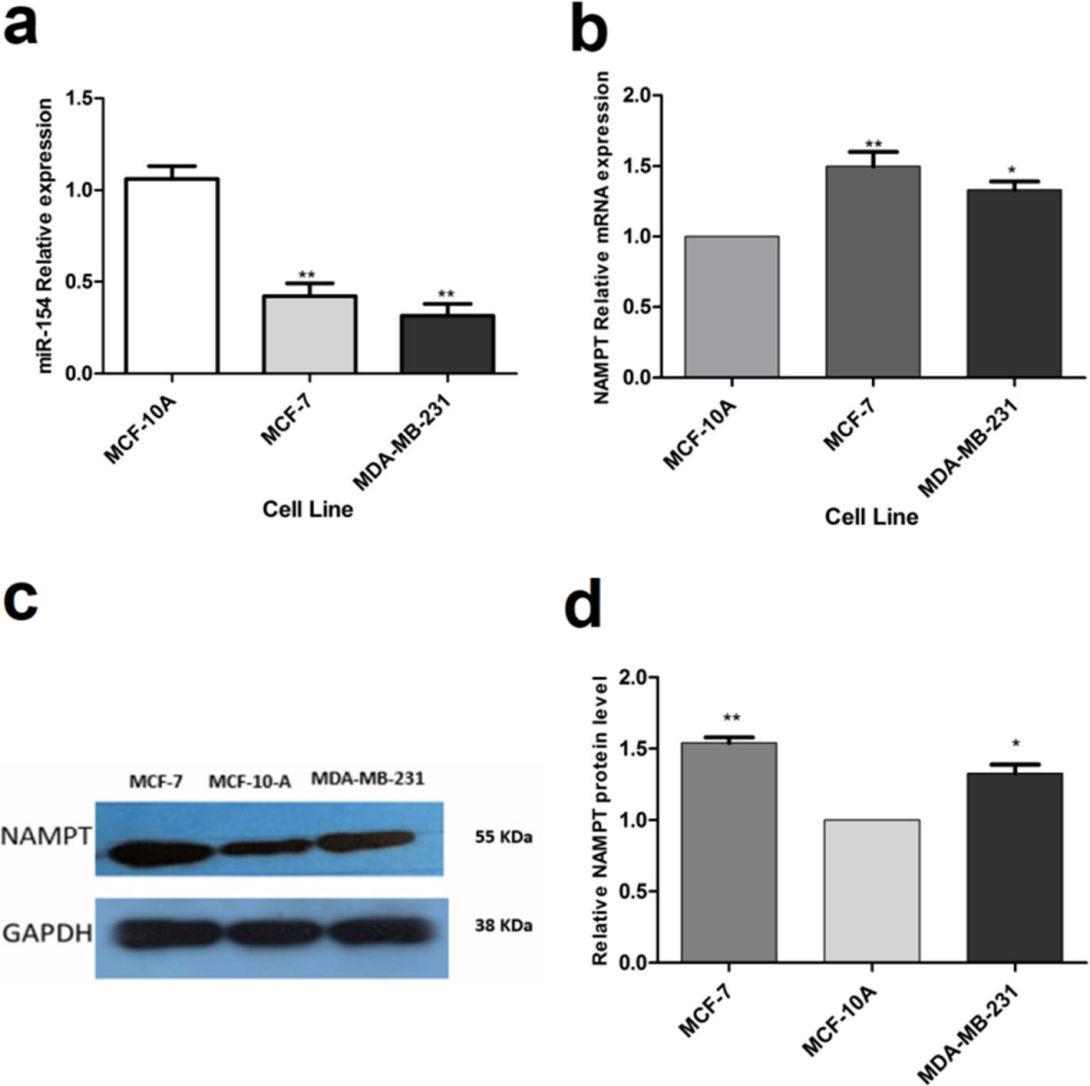
The expression level of miR-154 and NAMPT in un-transfected cells. Basal expression levels of (**a**) miR-154 and (**b**) NAMPT were compared with those in MCF-10A cells. Each vertical bar represents the mean ± SD of triplicate determinations. *P < 0.05; **P < 0.01. (**c**) Evaluation of NAMPT basal expression at protein level by immunoblotting and (**d**) the quantification of the resulting bands by densitometry. Each bar is the mean ± SD of at least three independent experiments. *P < 0.05; **P < 0.01

Figure 1a shows the relative expression of miR-154 in untreated MDA-MB-231 and MCF-7 cell lines compared to normal epithelial cell line (MCF-10A) that was used as control. It can be observed that miR-154 expression levels were considerably lower in MDA-MB-231 and MCF-7 (both *P* < 0.01) cell lines in comparison with MCF-10A cells. Additionally, measurement of the mRNA expression of NAMPT indicated that NAMPT was expressed higher in MDA-MB-231 (*P* < 0.05) and MCF-7 (*P* < 0.01) cell lines as opposed to MCF-10A (Fig. 1b). Furthermore, the blotting results showed that the basal level of NAMPT protein in MDA-MB-231 and MCF-7 and cell lines was higher than MCF-10A (*P* values less than 0.05 and 0.01, respectively) (Fig. 1 c, d).

### miR-154 cellular levels was up-regulated via miRNA mimic transfection

**Fig. 2:**
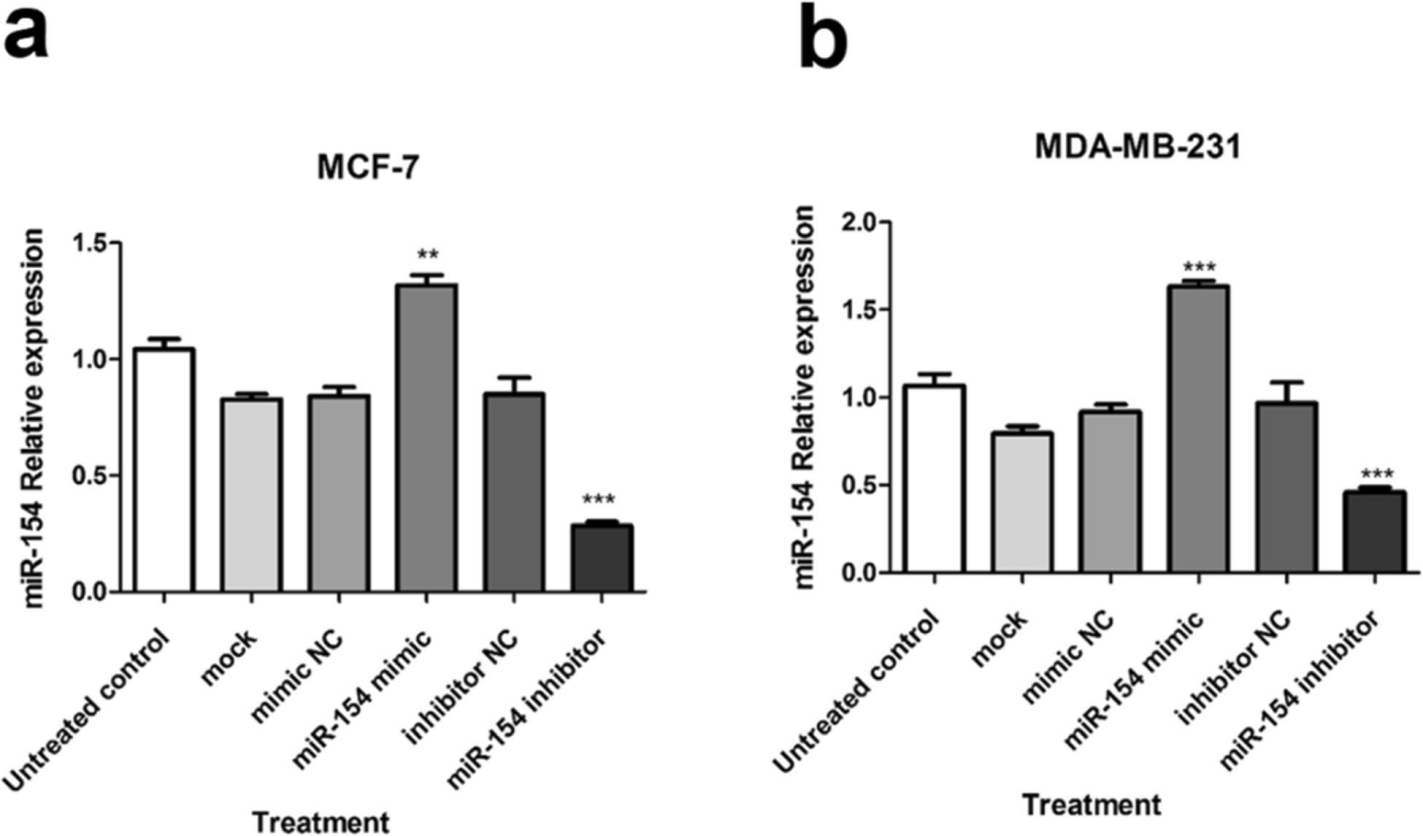
Relative expression of miR-154 after transfection of breast cancer cells. The cellular level of miR-154 after transfection of (**a**) MCF-7 and (**b**) MDA-MB-231 cells with different miR-154-related oligonucleotides compared to untreated control cells. **P < 0.01, *** *P* < 0.001

In order to clarify the mechanism by which miR-154 controls NAMPT expression, transfection experiments were conducted. Cellular transfection was conducted with either miR-154 mimic which was expected to increase the intracellular levels of miR-154 or its antisense oligonucleotide serving as miR-154 inhibitor to sequester or decrease miR-154. The MCF-7 cell line transfected with the mimic showed a significant increase in miR-154 levels (*P* < 0.01), while, a decline in miR-154 expression was observed following transfection with its inhibitor (*P* < 0.001) (Fig. 2a). The MDA-MB-231 cells also exhibited a significantly enhanced cellular levels of miR-154 after transfection with miRNA-mimic (*P* < 0.001). In contrast, transfection of MDA-MB-231 cells with miR-154 inhibitor was associated with a remarkable decrease in miR-154 level (*P* < 0.001) (Fig. 2b). Fluorescence microscopy results of the cells transfected with fluorescein-labeled microRNAs confirmed successful transfection (Additional file 1: Figure S1).

### miR-154 and NAMPT gene expression

**Fig. 3:**
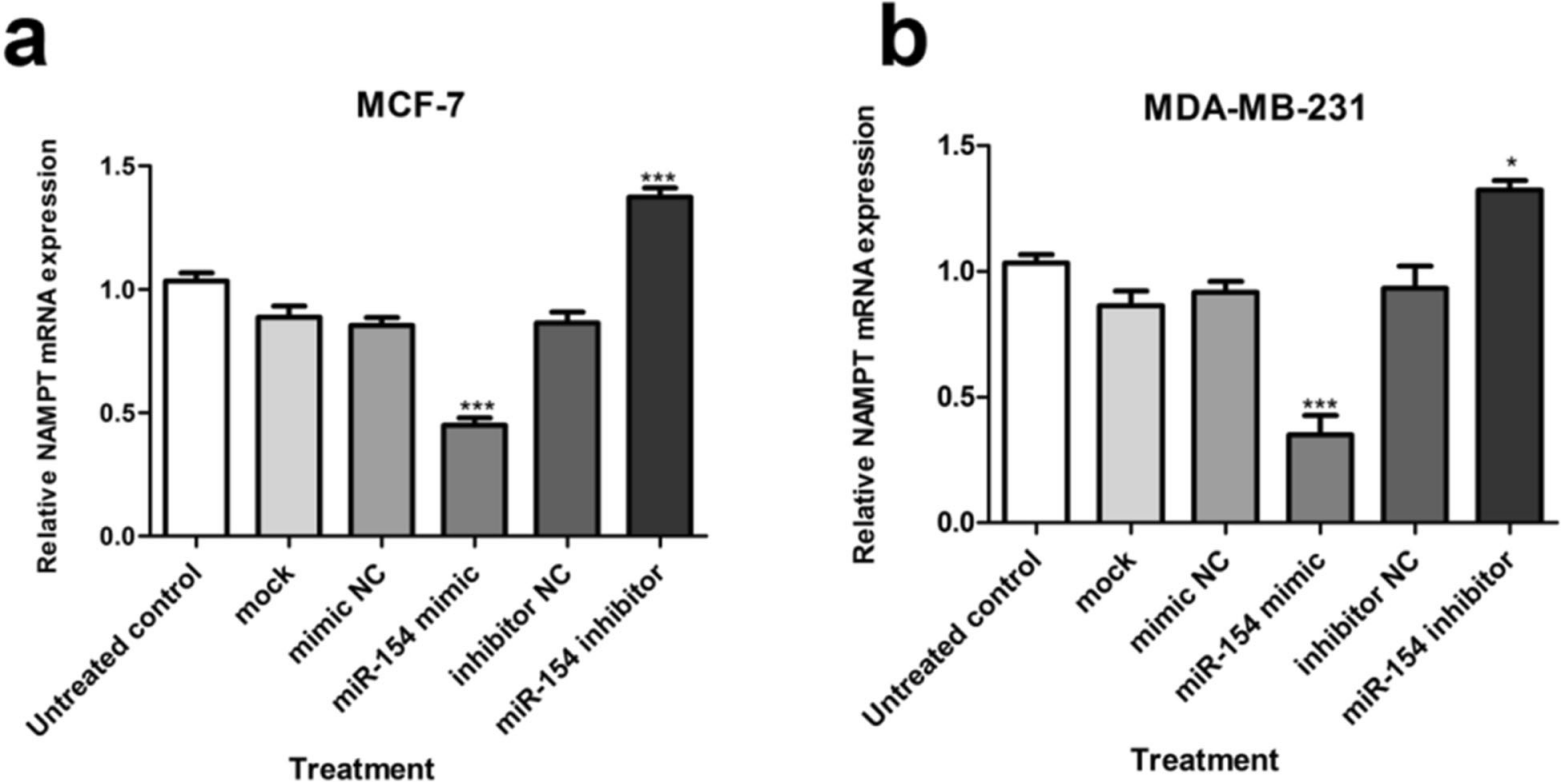
*NAMPT* gene expression in breast cancer cells after transfection. Relative NAMPT mRNA expression in (**a**) MCF-7 and (**b**) MDA-MB-231 cells transfected with miR-154 mimic, miR-154 inhibitor or their negative controls (NC) compared to untreated cells. Each column represents the mean ± SD of at least three separate experiments. *P < 0.05; ***P < 0.001

As described earlier, bioinformatics analysis anticipated that 3′-UTR of NAMPT is potentially targeted by miR-154. So, it was supposed that down-regulated miR-154 in cancer cells might be involved in NAMPT up-regulation. To evaluate whether miR-154 would exert an inhibitory effect on NAMPT expression, RT-PCR were performed on transfected human breast cancer cells. At the mRNA level, *NAMPT* gene revealed a significantly reduced expression in both breast cancer cell lines (*P <* 0.001) due to miR-154 augmentation by its mimic. Quite the reverse, blocking miR-154 by its corresponding inhibitor caused a significant increase in the expression of NAMPT mRNA in both of the studied cell lines (*P <* 0.001 and *P <* 0.05, respectively) (Fig. 3a, b).

### Suppression of NAMPT protein expression by miR-154

**Fig. 4:**
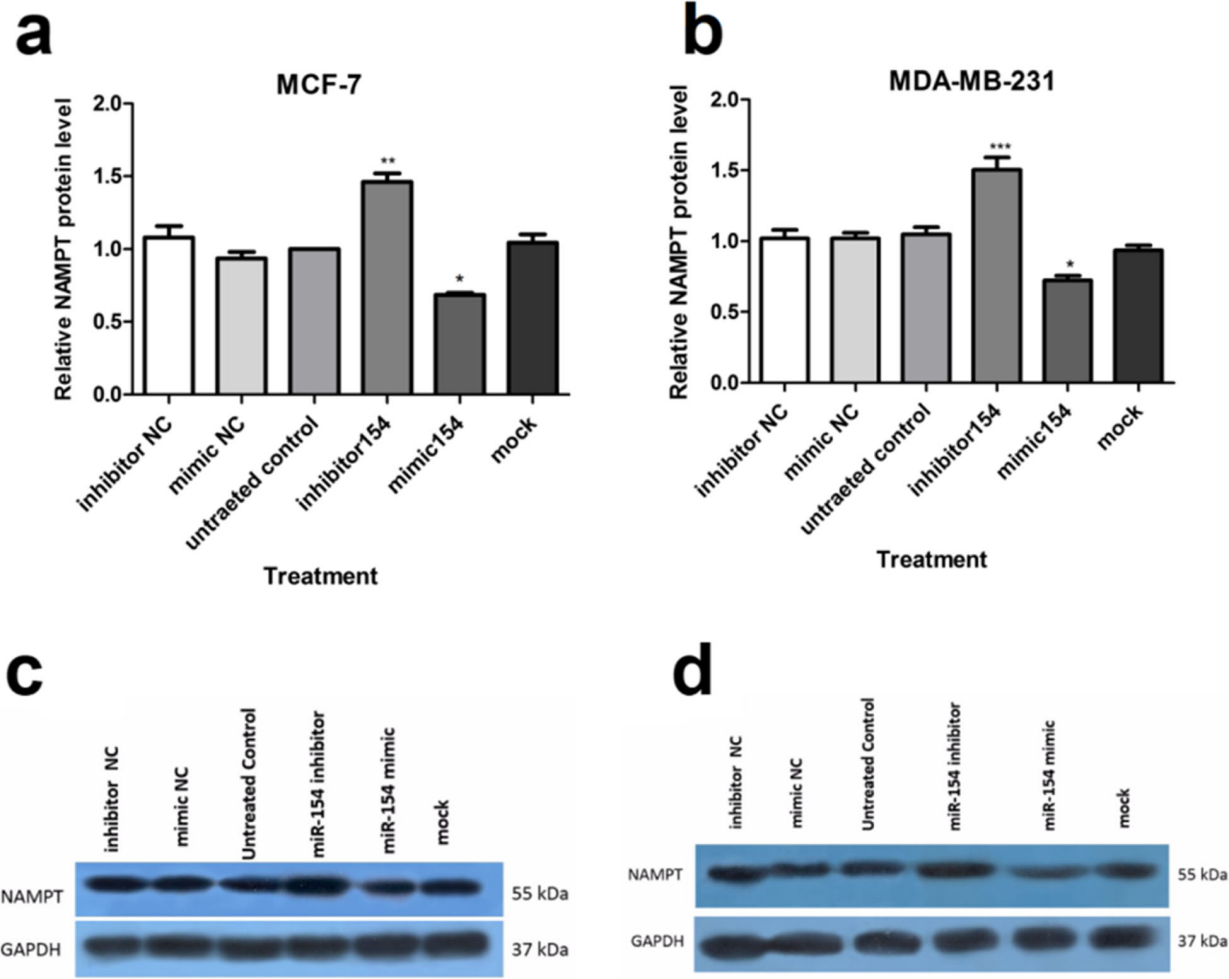
Suppression of NAMPT protein expression by miR-154. Quantitation of NAMPT protein level in (**a**) MCF-7 and (**b**) MDA-MB-231 cells transfected with the mimic of miR-154 or its inhibitor. Negative controls (NC) were also used for transfection. The results were compared to untreated control. Graphs represent the mean ± SD of the results of the densitometric analysis of the blotting images normalized to GAPDH as the internal control and presented relative to those in control cells. Representative immunoblot images of NAMPT protein measurement in (**c**) MCF-7 and (**d**) MDA-MB-231 cells. *P < 0.05, **P < 0.01, *** *P* < 0.001

The results obtained from Western blotting experiments indicated that the up-regulation of miR-154 via transfection with miR-154 mimic, remarkably reduced the levels of NAMPT protein in MCF-7 (P < 0.05) as well as MDA-MB-231 (P < 0.05) cells (Fig. 4a, b). Nevertheless, NAMPT protein expression was enhanced in both MCF-7 (*P* < 0.01) and MDA-MB-231 (*P* < 0.001) cell lines following transfection with miR-154 inhibitor (Fig. 4a, b).

### The effect of miR-154 on NAD depletion

**Fig. 5:**
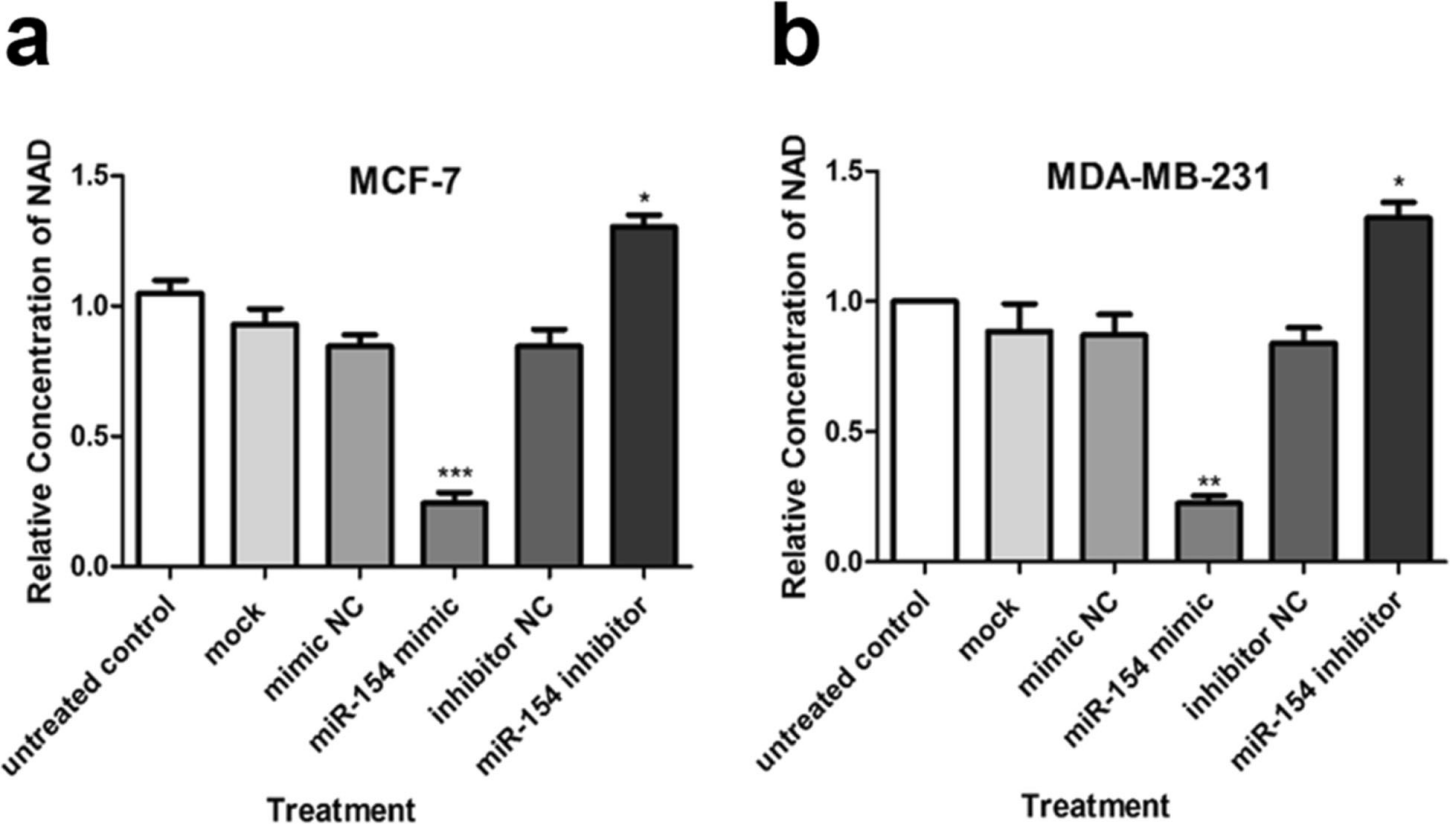
The effect of miR-154 on intracellular NAD levels. Evaluation of relative NAD levels in (**a**) MCF-7 and (**b**) MDA-MB-231 cell lines after transfection with miR-154 mimic, inhibitor or their negative controls (NC) compared to un-transfected control. Results are presented as mean ± SD from three duplicate experiments that were performed separately. *P < 0.05, ** *P* < 0.01, ***P < 0.001

Increased NAMPT level is correlated with high concentration of NAD in malignant cells [5]. Our results showed that NAD was decreased in the MCF-7 cells that were transfected with the mimic of miR-154 compared to un-transfected control cells (*P* < 0.001). On the contrary, there was a significant augmentation of NAD level in the cells that were transfected with miR-154 inhibitor (*P* < 0.05) (Fig. 5a). Similarly, the NAD level in MDA-MB-231 cells transfected with the mimic of miR-154 exhibited a significant increase (*P* < 0.01), while a considerable decrease in NAD was observed in those transfected with miR-154 inhibitor (*P* < 0.05) (Fig. 5b).

### Increase of miR-154 in breast cancer cells reduced cell viability

**Fig. 6:**
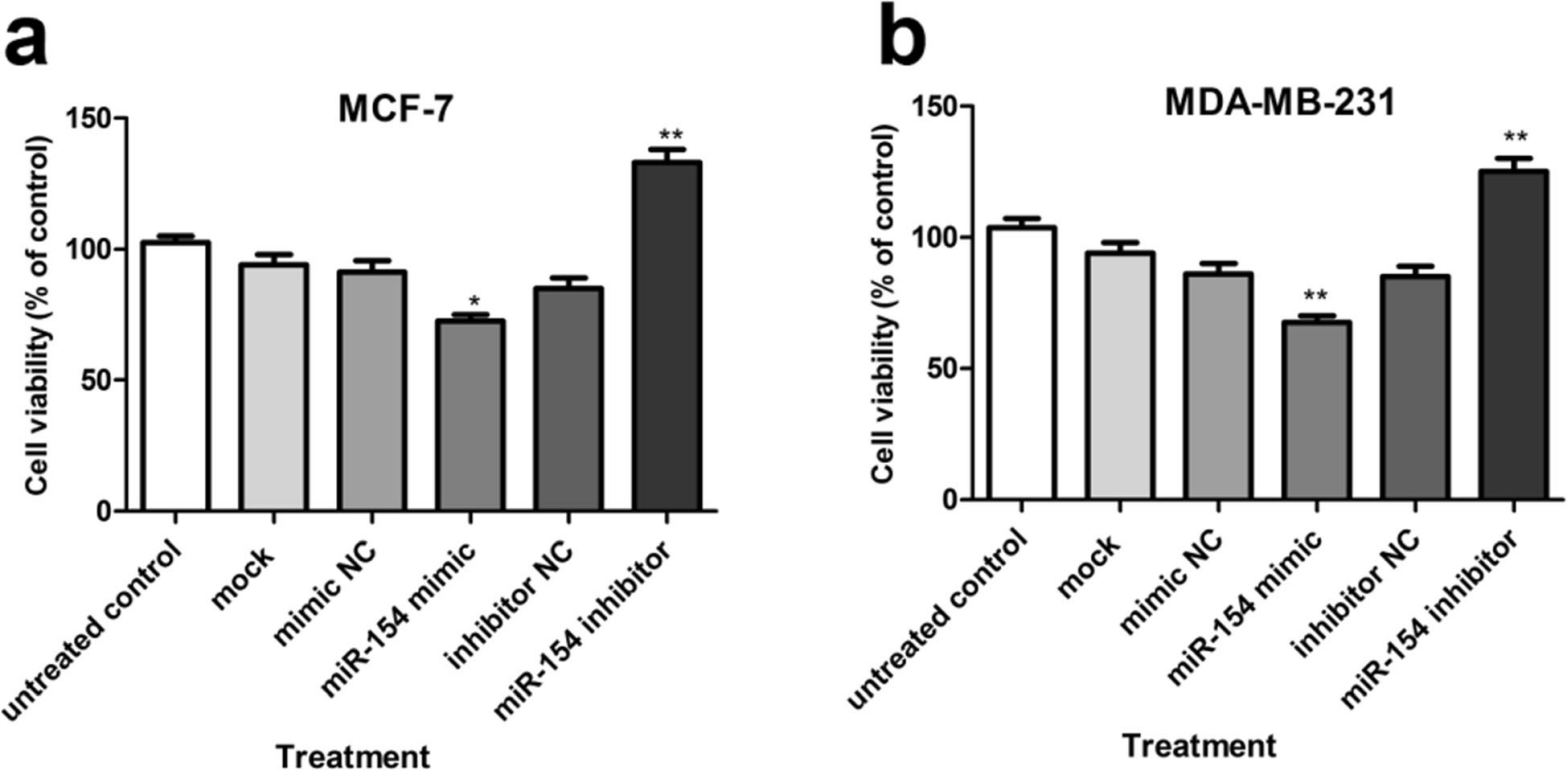
WST-1 cell survival assay. Survival of (**a**) MCF-7 and (**b**) MDA-MB-231 cells in response to increased and decreased levels of miR-154 by its mimic and inhibitor, respectively. The obtained results are expressed as percentage to untreated control. Data are mentioned as mean ± SD of triplicate experiments that were repeated at least three times. * *P* < 0.05, ** *P* < 0.01

NAMPT is elevated in diverse human malignancies such as breast cancer. This enzyme facilitates proliferation and increases survival of cancer cells [22]. In the present research, we studied the effect of miR-154 on the survival of breast cancer cells using WST-1 cell survival assay. The obtained results revealed that miR-154 mimic considerably reduced cell survival in MCF-7 (*P* < 0.05) and MDA-MB-231 (*P* < 0.01) cells when compared to the untreated cells; whereas, treating the cells with miR-154 inhibitor considerably enhanced cell survival in both cell lines (both *P* < 0.01). The obtained results are shown in Fig. 6.

### miR-154 increased the susceptibility of breast cancer cells to doxorubicin

**Fig. 7:**
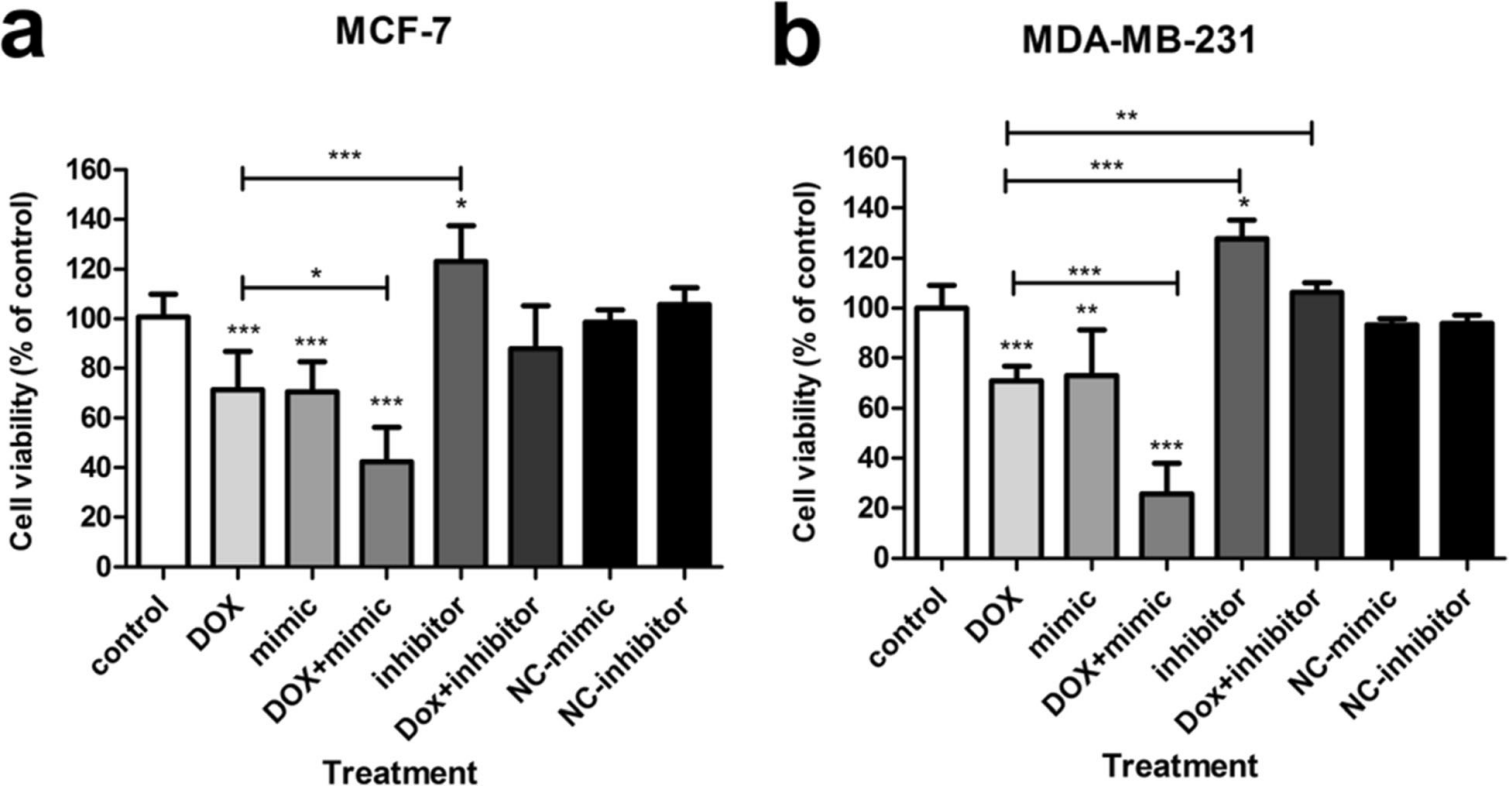
Effect of miR-154 on susceptibility of breast cancer cells to doxorubicin. The effect of doxorubicin (DOX) either alone or in combination with miR-154 oligonucleotides on the viability of (**a**) MCF-7 and (**b**) MDA-MB-231 breast cancer cells. The results are presented as mean ± SD, relative to un-transfected controls. * *P* < 0.05, ** *P* < 0.01, *** *P* < 0.001

Considering the effect of miR-154 on cell viability, we treated the studied cell lines with doxorubicin after transfection. As it is shown in Fig. 7, when miR-154 mimic was used in combination with doxorubicin, the cell viability was significantly diminished compared to either doxorubicin or miR-154 mimic alone. This effect was observed in both MCF-7 and MDA-MB-231 cells (Fig. 7 a, b). On the contrary, down-regulation of cellular miR-154 by its inhibitor led to a lower response to doxorubicin treatment and the cell viability in this group (miR-154 inhibitor + doxorubicin) was similar to untreated control cells (Fig. 7).

### Up-regulation of miR-154 promoted apoptosis in breast cancer cells

**Fig. 8:**
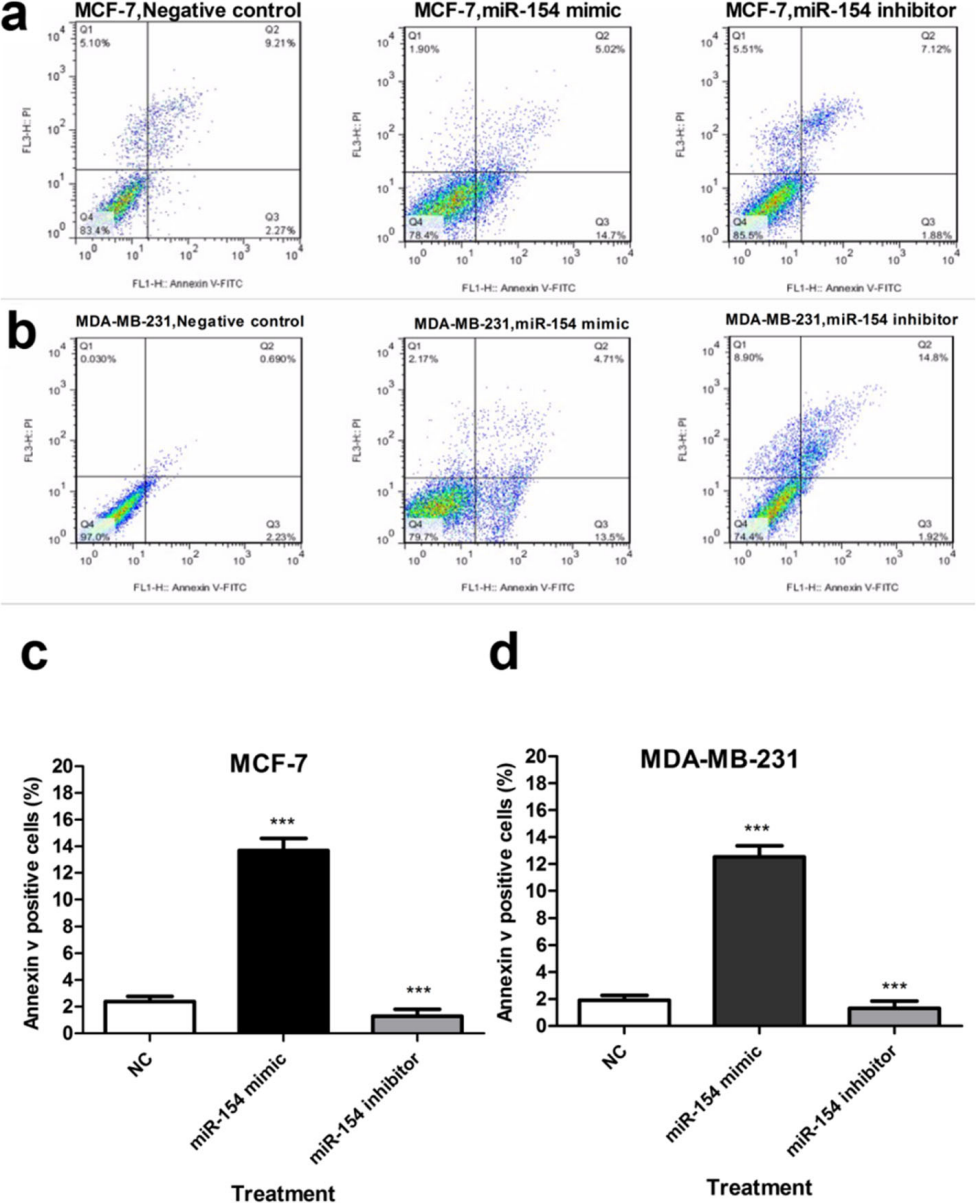
Cell apoptosis assay using Annexin V and propidium iodide. A quadrant dot plot of the results of flow cytometry assay after Annexin V/PI staining and average percentage of apoptotic cells in (**a**) MCF-7 and (**b**) MDA-MB-231 cells treated with miR-154 mimic or inhibitor. The apoptotic cells were exhibited as the blue dots in the lower right quadrant of each diagram. The diagram showing the quantification of the percentage of apoptotic cells in MCF-7 (**c**) and MDA-MB-231 cells (**d**). The obtained results were compared to the untransfected controls and are presented as mean ± SD. *** *P* < 0.001

The results of flow cytometry analysis revealed that transfection with the mimic of miR-154 significantly induced apoptosis in MCF-7 and MDA-MB-231 cells (both *P* < 0.001). On the contrary, down-regulation of miR-154 by its inhibitor decreased cell death percentage in MCF-7 and MDA-MB-231 cells (both *P* < 0.001) (Fig. 8).

### miR-154 regulated NAMPT by direct binding to its 3′-UTR

**Fig. 9:**
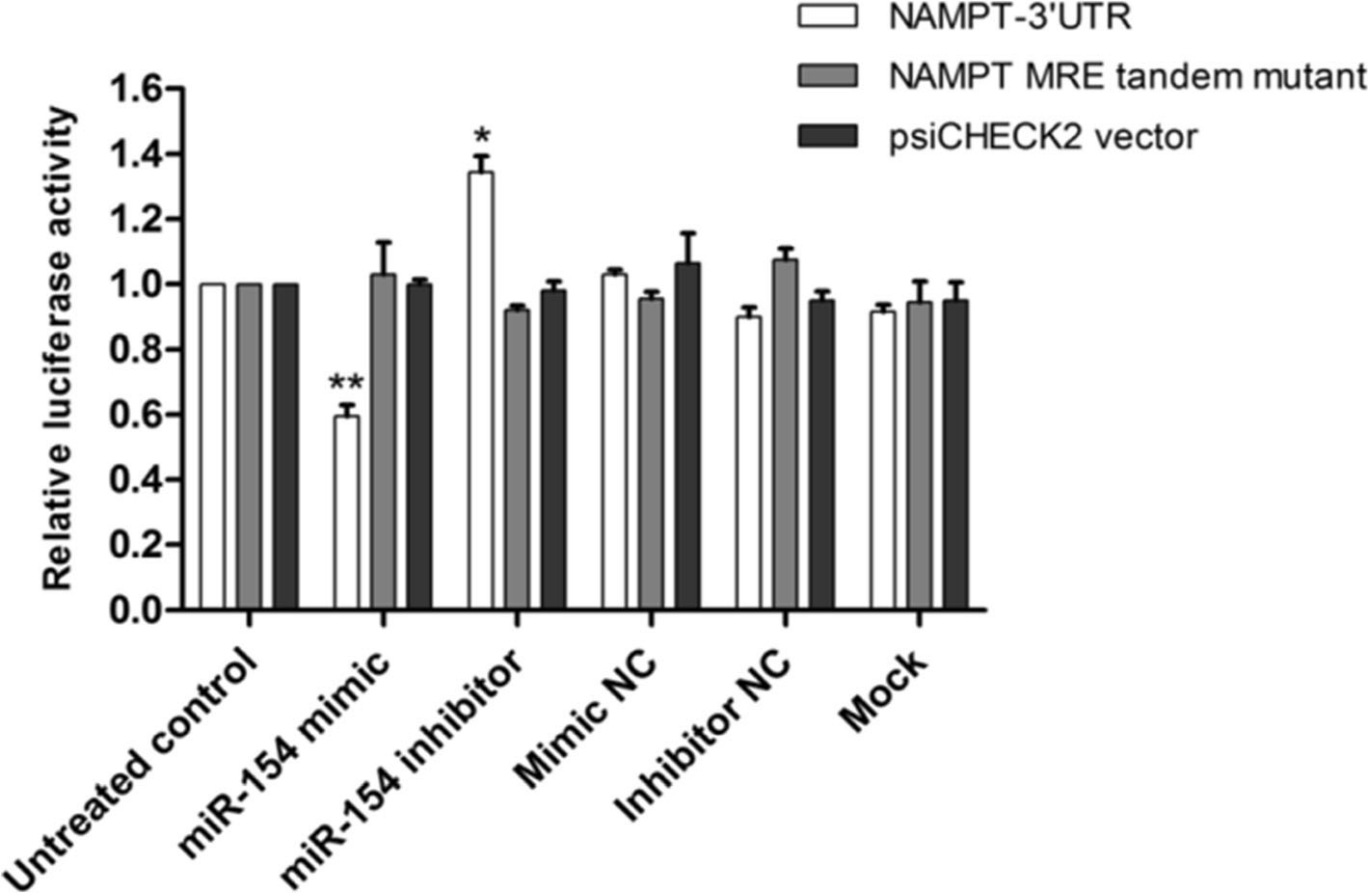
Luciferase reporter assay verifying the predicted interaction between miR-154 and 3′-UTR of NAMPT. PsiCHECK2 vector harboring NAMPT 3′-UTR or the mutant form of miR-154 recognition element (NAMPT-MRE-tandem-mut) were co-transfected with microRNA-related oligonucleotides into HEK293T. Firefly luciferase activity was normalized with respect to Renilla luciferase as control. The experiments were repeated at least three times. * *P* < 0.05, ** *P* < 0.01

As previously stated, bioinformatics analysis showed that miR-154 is among the miRNAs that are conserved among mammals and it was predicted that 3′-UTR region of NAMPT mRNA could be a potential target for miR-154. To confirm this, the luciferase reporter activity of psiCHECK2 vector having NAMPT-related 3′-UTR in the presence of desired oligonucleotides was investigated. miR-154 mimic decreased the luciferase activity by 59.5 ± 0.03% compared to untreated control cells (*P* < 0.01); however, miR-154 inhibitor led to a significant increase in luciferase activity (*P* < 0.05) (Fig. 9). None of the controls significantly affected the luciferase activity.

## Discussion

Over 2 million new cases of breast cancer have been reported in 2018 “(https://www.wcrf.org/dietandcancer/cancer-trends/breast-cancer-statistics)”. Therefore, novel methods including strategies based on cancer-related molecular changes are essential for breast cancer management. In this research, we indicated that the miR-154 expression was remarkably reduced in breast cancer cell lines in comparison with normal mammary cells. Consistently, Qin et al. studied the role of miR-154 in breast carcinogenesis and showed that miR-154 was down-regulated in this malignancy and was able to decrease cellular proliferation and metastasis potential through targeting ADAM metallopeptidase domain 9 (ADAM9) [23]. In another study by Xu et al., E2F transcription factor 5 protein (E2F5) was introduced as a direct target of miR-154, with reciprocal relationship between these two parameters in in neoplastic cells of breast [14]. Additionally, reduced levels of miR-154 and its association with aggressive clinicopathological characteristics have been reported in glioma, colorectal, prostate and non-small cell lung cancers [24–27].

In the current study, a negative correlation was found between miR-154 and NAMPT expression (under- and over-expressed, respectively) in breast cancer cells, suggesting the inhibitory effect of miR-154 on NAMPT expression. Significantly higher expression of NAMPT in breast cancer tissues compared with normal mammary gland tissue has been previously reported and has been shown to be related to a higher tumor growth, advanced clinical stages, increased expression of progesterone and estrogen receptors and lymph node metastasis. Meanwhile, over expression of NAMPT in patients results in poor overall or disease-free survival [21, 22, 28–32]. Additionally, exogenous administration of recombinant NAMPT not only leads to increased cell proliferation by activation of signaling pathways, but also increases cell survival by NAD production [33, 34].

The results achieved in our study explained that the up-regulation of miR-154 significantly suppressed NAMPT expression both at mRNA and protein levels; indicating that this interaction leads to mRNA degradation or suppression of translation as has been suggested as mechanisms of action of miRNAs [26]. The regulatory effect of miR-154 was further confirmed by reducing its cellular levels using miR-154 inhibitor which in turn led to a significant rise in NAMPT expression levels. The obtained results from luciferase reporter assay demonstrated that the aforementioned regulatory outcome of miR-154 on NAMPT was directly the effect of binding of miR-154 to the NAMPT 3′-UTR, ruling out the possibility of off-targets and indirect effects. These findings are consistent with our recent study in which, miR-206 was introduced as a potential inhibitor of NAD biosynthesis in HBC cells via direct binding to NAMPT 3′-UTR [21]. Consistently, NAMPT 3′-UTR has been reported in a number of studies, as the potential target for a wide variety of miRNAs in other malignancies and diseases including miR-182 in the ossification of ligamentum flavum [35], miR-300 in neonatal sepsis [36], miR-34a in obesity [37], miR-206 in pancreatic cancer [38], miR-26b in colorectal cancer [39], miR-410 in pulmonary arterial hypertension (PAH) [40] and miR-182 in HIV-1 contaminated cells [41].

The findings of NAD measurement revealed that miR-154 caused the attenuation of intracellular NAD via inhibition of NAMPT salvage pathway. NAMPT is an essential enzyme in NAD biosynthesis and therefore its inhibition is a plausible approach in depleting intracellular NAD [22, 42]. Here we showed that inhibition of NAMPT and the further decline in NAD levels leads to diminished cell viability and a prominent induction of apoptosis. Earlier, we had reported that inhibition of NAMPT by its specific inhibitor could effectively reduce NAD levels and induce apoptosis in breast cancer cells [43]. In line with our findings, inhibition of NAMPT by microRNAs has also been shown to be an appropriate method in modulating NAD content and cell viability [37–39].

One major obstacle in cancer therapy is chemoresistance to the commercial cancer therapeutic drugs, contributing to cancer progression, recurrence and mortality [44]. Aberrant miRNA expression is related to anti-cancer drug resistance [44]. In the current study, we revealed that miR-154 increased the cellular response to DOX and cell survival was significantly decreased when DOX treatment was combined with up-regulation of miR-154. Consistently, up-regulation of miR-760 has been shown to be able to sensitize breast cancer cells to the anti-cancer drugs [45]. miR-133b has also been found to be functionally involved in the DOX resistant breast cancer cells and the intra-tumoral delivery of this miRNA increased the treatment response to DOX in DOX-resistant xenografts [46]. NAMPT overexpression has been shown to be associated with low response to chemotherapeutic agents including doxorubicin, etoposide, fluorouracil, paclitaxel, and phenylethyl isothiocyanate [6]. Thus, over-expression of miR-154 might be an efficacious approach to increase the sensitivity of breast cancer cells to DOX treatment.

## Conclusion

The obtained findings of this research suggest that the role of miR-154 in the inhibition of NAMPT-mediated NAD production pathway via targeting NAMPT 3′-UTR is crucial. Overexpression of this miRNA reduces cell survival, stimulates apoptosis, and increases the susceptibility of breast cancer cells to chemotherapy. As a result, this molecular mechanism can be employed as a promising method for breast cancer treatment.

## Supplementary information

**Additional file 1: Figure S1.** MCF-7 & MDA-MB-231 cell lines transfected with FAM-labeled microRNAs. Fluorescence microscopy images of MCF-7 and MDA-MB-231 cells transfected with miR-154 mimic and miR inhibitor negative controls (NC) labeled with FAM. a) Evaluation of transfection efficiency under fluorescence and light microscope in MCF-7 cells transfected with FAM-labeled miR-154 mimic NC and miR-inhibitor NC. b) Evaluation of transfection efficiency, under fluorescence and light microscope, in MDA-MB-231 cells transfected with FAM-labeled miR-154 mimic NC and miR-inhibitor NC. The fluorescence microscopy images were obtained 24 h after transfection. **Table S1.** primer sequences for real-time PCR, and primer sequences used for amplification and cloning of NAMPT 3′-UTR. The underlined sequences represent the restriction enzyme recognition sites (CTCGAG for XhoI and GCGGCCGC for NotI) added for cloning into psiCHECK-2.

## Data Availability

The datasets used and/or analysed during the current study are available from the corresponding author on reasonable request.

## Abbreviations

3′-UTR: 3′-untranslated region
BCA: bicinchoninic acid assay
CT: cholera toxin
DMEM: Dulbecco’s modified Eagle medium
DOX: Doxorubicin
EGF: epithelial growth factor
FBS: Fetal bovine serum
GAPDH: Glyceraldehyde 3-Phosphate Dehydrogenase
HBC: human breast cancer
MEGM: mammary epithelial cell growth medium
miRNA: microRNA
MRE: microRNA response element
NAD: nicotinamide adenine dinucleotide
NAMPT: nicotinamide phosphoribosyltransferase
NC: negative control.
PAP: Poly (A) Polymerase
PARP1: poly ADP-ribose polymerase 1
PEI: polyethylenimine
PMSF: phenylmethane sulfonyl fluoride
PVDF: polyvinylidine difluoride membrane

## References

[1] Jafari SH, Saadatpour Z, Salmaninejad A, Momeni F, Mokhtari M, Nahand JS (2018). Breast cancer diagnosis: imaging techniques and biochemical markers. J Cell Physiol.

[2] Ju J, Zhu AJ, Yuan P (2018). Progress in targeted therapy for breast cancer. Chronic diseases and translational medicine.

[3] Vranic S, Palazzo J, Sanati S, Florento E, Contreras E, Xiu J, et al. Potential novel therapy targets in neuroendocrine carcinomas of the breast. Clinical breast cancer. 2018. 10.1016/j.clbc.2018.09.001 30268765

[4] Grolla AA, Travelli C, Genazzani AA, Sethi JK (2016). Extracellular nicotinamide phosphoribosyltransferase, a new cancer metabokine. Br J Pharmacol.

[5] Shackelford RE, Mayhall K, Maxwell NM, Kandil E, Coppola D (2013). Nicotinamide phosphoribosyltransferase in malignancy: a review. Genes & cancer.

[6] Sampath D, Zabka TS, Misner DL, O'Brien T, Dragovich PS (2015). Inhibition of nicotinamide phosphoribosyltransferase (NAMPT) as a therapeutic strategy in cancer. Pharmacol Ther.

[7] Menssen A, Hydbring P, Kapelle K, Vervoorts J, Diebold J, Lüscher B (2012). The c-MYC oncoprotein, the NAMPT enzyme, the SIRT1-inhibitor DBC1, and the SIRT1 deacetylase form a positive feedback loop. Proc Natl Acad Sci.

[8] Zangooei M, Nourbakhsh M, Ghahremani MH, Meshkani R, Khedri A, Shadboorestan A (2018). Investigating the effect of visfatin on ERalpha phosphorylation (Ser118 and Ser167) and ERE-dependent transcriptional activity. EXCLI J.

[9] Chini CC, Guerrico AM, Nin V, Camacho-Pereira J, Escande C, Barbosa MT (2014). Targeting of NAD metabolism in pancreatic cancer cells: potential novel therapy for pancreatic tumors. Clinical cancer research : an official journal of the American Association for Cancer Research.

[10] Folgueira MA, Carraro DM, Brentani H, Patrao DF, Barbosa EM, Netto MM (2005). Gene expression profile associated with response to doxorubicin-based therapy in breast cancer. Clinical cancer research : an official journal of the American Association for Cancer Research.

[11] Noruzi S, Azizian M, Mohammadi R, Hosseini SA, Rashidi B, Mohamadi Y, et al. Micro-RNAs as critical regulators of matrix metalloproteinases in cancer. Journal of cellular biochemistry. 2018. 10.1002/jcb.27182 30132957

[12] Keshavarz M, Dianat-Moghadam H, Sofiani VH, Karimzadeh M, Zargar M, Moghoofei M (2018). miRNA-based strategy for modulation of influenza a virus infection. Epigenomics..

[13] Wang W, Luo YP (2015). MicroRNAs in breast cancer: oncogene and tumor suppressors with clinical potential. J Zhejiang Univ Sci B.

[14] Xu H, Fei D, Zong S, Fan Z (2016). MicroRNA-154 inhibits growth and invasion of breast cancer cells through targeting E2F5. Am J Transl Res.

[15] Wang W, Peng B, Wang D, Ma X, Jiang D, Zhao J (2011). Human tumor microRNA signatures derived from large-scale oligonucleotide microarray datasets. Int J Cancer.

[16] Zhou H, Zhang M, Yuan H, Zheng W, Meng C, Zhao D (2016). MicroRNA-154 functions as a tumor suppressor in osteosarcoma by targeting Wnt5a. Oncol Rep.

[17] Pang X, Huang K, Zhang Q, Zhang Y, Niu J (2015). miR-154 targeting ZEB2 in hepatocellular carcinoma functions as a potential tumor suppressor. Oncol Rep.

[18] Mian C, Pennelli G, Fassan M, Balistreri M, Barollo S, Cavedon E (2012). MicroRNA profiles in familial and sporadic medullary thyroid carcinoma: preliminary relationships with RET status and outcome. Thyroid : official journal of the American Thyroid Association.

[19] Xin C, Zhang H, Liu Z (2014). miR-154 suppresses colorectal cancer cell growth and motility by targeting TLR2. Mol Cell Biochem.

[20] Lin X, Yang Z, Zhang P, Shao G (2015). miR-154 suppresses non-small cell lung cancer growth in vitro and in vivo. Oncol Rep.

[21] Hesari Z, Nourbakhsh M, Hosseinkhani S, Abdolvahabi Z, Alipour M, Tavakoli-Yaraki M, et al. Down-regulation of NAMPT expression by mir-206 reduces cell survival of breast cancer cells. Gene. 2018. 10.1016/j.gene.2018.06.021 29886033

[22] Zhou SJ, Bi TQ, Qin CX, Yang XQ, Pang K (2018). Expression of NAMPT is associated with breast invasive ductal carcinoma development and prognosis. Oncol Lett.

[23] Qin C, Zhao Y, Gong C, Yang Z (2017). MicroRNA-154/ADAM9 axis inhibits the proliferation, migration and invasion of breast cancer cells. Oncol Lett.

[24] Kai Y, Qiang C, Xinxin P, Miaomiao Z, Kuailu L (2015). Decreased miR-154 expression and its clinical significance in human colorectal cancer. World journal of surgical oncology.

[25] Qiao W, Cao N, Yang L (2017). MicroRNA-154 inhibits the growth and metastasis of gastric cancer cells by directly targeting MTDH. Oncol Lett.

[26] Wang L, Wu L, Wu J (2016). Downregulation of miR-154 in human glioma and its clinicopathological and prognostic significance. J Int Med Res.

[27] Liu S, Yang Y, Chen L, Liu D, Dong H (2018). MicroRNA-154 functions as a tumor suppressor in non-small cell lung cancer through directly targeting B-cell-specific Moloney murine leukemia virus insertion site 1. Oncol Lett.

[28] Hong S, Park C, Kim S, Nam Y, Yu J, Shin J (2016). NAMPT suppresses glucose deprivation-induced oxidative stress by increasing NADPH levels in breast cancer. Oncogene..

[29] Kim JG, Kim EO, Jeong BR, Min YJ, Park JW, Kim ES (2010). Visfatin stimulates proliferation of MCF-7 human breast cancer cells. Mol Cell.

[30] Lee Y-C, Yang Y-H, Su J-H, Chang H-L, Hou M-F, Yuan S-SF. High visfatin expression in breast cancer tissue is associated with poor survival. Cancer Epidemiology and Prevention Biomarkers. 2011. 10.1158/1055-9965.EPI-11-0399 21784959

[31] Zhou T, Wang T, Garcia JG (2014). Expression of nicotinamide phosphoribosyltransferase-influenced genes predicts recurrence-free survival in lung and breast cancers. Sci Rep.

[32] Park H, Lee MJ, Jeong JY, Choi MC, Jung SG, Joo WD (2014). Dysregulated microRNA expression in adenocarcinoma of the uterine cervix: clinical impact of miR-363-3p. Gynecol Oncol.

[33] Gholinejad Zafar, kheiripour Nejat, Nourbakhsh Mitra, Ilbeigi Davod, Behroozfar Kiarash, Hesari Zahra, Golestani Abolfazl, Shabani Mohammad, Einollahi Nahid (2017). Extracellular NAMPT/Visfatin induces proliferation through ERK1/2 and AKT and inhibits apoptosis in breast cancer cells. Peptides.

[34] Behrouzfar K, Alaee M, Nourbakhsh M, Gholinejad Z, Golestani A (2017). Extracellular NAMPT/visfatin causes p53 deacetylation via NAD production and SIRT1 activation in breast cancer cells. Cell Biochem Funct.

[35] Zhang Q, Shen Y, Jiang Y, Zhao S, Zhou D, Xu N (2018). Overexpression of miR-182 inhibits ossification of ligamentum flavum cells by targeting NAMPT. Exp Cell Res.

[36] Li Y, Ke J, Peng C, Wu F, Song Y (2018). MicroRNA-300/NAMPT regulates inflammatory responses through activation of AMPK/mTOR signaling pathway in neonatal sepsis. Biomed Pharmacother.

[37] Choi SE, Fu T, Seok S, Kim DH, Yu E, Lee KW (2013). Elevated microRNA-34a in obesity reduces NAD+ levels and SIRT1 activity by directly targeting NAMPT. Aging Cell.

[38] Ju H-Q, Zhuang Z-N, Li H, Tian T, Lu Y-X, Fan X-Q (2016). Regulation of the Nampt-mediated NAD salvage pathway and its therapeutic implications in pancreatic cancer. Cancer Lett.

[39] Zhang C, Tong J, Huang G (2013). Nicotinamide phosphoribosyl transferase (Nampt) is a target of microRNA-26b in colorectal cancer cells. PLoS One.

[40] Gao H, Chen J, Chen T, Zhao S, Machado RF. Microrna-410 Is Downregulated By Hypoxia And VEGF Inhibits Proliferation Of Pulmonary Artery Smooth Muscle Cells And Pulmonary Artery Endothelial Cells Via Regulation Of Nicotinamide Phosphoribosyl Transferase. B71 PULMONARY HYPERTENSION LIFE: ANIMAL MODELS AND EX VIVO STUDIES IN PULMONARY HYPERTENSION: American Thoracic Society; 2017. p. A4217-A.

[41] Chen X-Y, Zhang H-S, Wu T-C, Sang W-W, Ruan Z (2013). Down-regulation of NAMPT expression by miR-182 is involved in tat-induced HIV-1 long terminal repeat (LTR) transactivation. Int J Biochem Cell Biol.

[42] Sawicka-Gutaj N, Waligórska-Stachura J, Andrusiewicz M, Biczysko M, Sowiński J, Skrobisz J (2015). Nicotinamide phosphorybosiltransferase overexpression in thyroid malignancies and its correlation with tumor stage and with survivin/survivin DEx3 expression. Tumor Biol.

[43] Alaee M, Khaghani S, Behroozfar K, Hesari Z, Ghorbanhosseini SS, Nourbakhsh M (2017). Inhibition of nicotinamide phosphoribosyltransferase induces apoptosis in estrogen receptor-positive MCF-7 breast cancer cells. J Breast Cancer.

[44] Allen KE, Weiss GJ (2010). Resistance may not be futile: microRNA biomarkers for chemoresistance and potential therapeutics. Mol Cancer Ther.

[45] Hu S, Wang C, Huang Z, Liu F, Xu C, Li X (2016). miR-760 mediates chemoresistance through inhibition of epithelial mesenchymal transition in breast cancer cells. Eur Rev Med Pharmacol Sci.

[46] Yuan Y, Yao YF, Hu SN, Gao J, Zhang L-L (2015). MiR-133a is functionally involved in doxorubicin-resistance in breast cancer cells MCF-7 via its regulation of the expression of uncoupling protein 2. PLoS One.

