# Supplementary material for: Suppression of nicotinamide phosphoribosyltransferase expression by miR-154 reduces the viability of breast cancer cells and increases their susceptibility to doxorubicin
Source: BMC Cancer. 2019 Nov 1;19:1027. doi: 10.1186/s12885-019-6221-0 (PMC6824125; doi:10.1186/s12885-019-6221-0)

Supplementary additional file 1

Supplementary Table 1

primer sequences for real-time PCR, and primer sequences used for amplification and cloning of NAMPT 3'-UTR. The underlined sequences represent the restriction enzyme recognition sites (CTCGAG for XhoI and GCGGCCGC for NotI) added for cloning into psiCHECK-2.

Template	Primer	Sequence (5′→3′)
	**MiRNA**	
**miR-154**	Forward	GGTTATCCGTGTTGCCTTCG
	Universal Reverse	GCGAGCACAGAATTAATACGACTC
**U6-snRNA (Internal Control)**	Forward	CTCGCTTCGGCAGCACA
	Reverse	AACGCTTCACGAATTTGCGT
**Reverse Transcription**		GCGAGCACAGAATTAATACGACTCACTATAGGTTTTTTTTTTTAG
	**Genes**	
**NAMPT**	Forward	GGTTCTTGGTGGAGGTTTGCTAC
	Reverse	GAAGACGTTAATCCCAAGGCC
**GAPDH (Internal Control)**	Forward	GGGAAGGTGAAGGTCGGAGT
	Reverse	TCCACTTTACCAGAGTTAAAAGCAG
**NAMPT-3′UTR**	Forward	CCGCTCGAGCGGGTACAGATGTGTGGGGTTTGTG
	Reverse	AAATATGCGGCCGCGCTGACATTCTCCACTGAATGGG
**NAMPT MRE**	Forward	CCGCTCGAGCGCTTTCACTTTCACTTCTCTT

**Tandem Mutant**		TTTCACTTTCACTTCTCTTCTCTCTTCCGTCC
	Reverse	ATAAGAATGCGGCCGCAGGGAGGGGAAAATGAGGACGGAAGAGAG

Supplementary Figure 1

MCF-7 & MDA-MB-231 cell lines transfected with FAM-labeled microRNAs .Fluorescence microscopy images of MCF-7 and MDA-MB-231 cells transfected with miR-154 mimic and miR inhibitor negative controls (NC) labeled with FAM. a) Evaluation of transfection efficiency under fluorescence and light microscope in MCF-7 cells transfected with FAM-labeled miR-154 mimic NC and miR-inhibitor NC. b) Evaluation of transfection efficiency, under fluorescence and light microscope, in MDA-MB-231 cells transfected with FAM-labeled miR-154 mimic NC and miR-inhibitor NC. The fluorescence microscopy images were obtained 24 h after transfection.

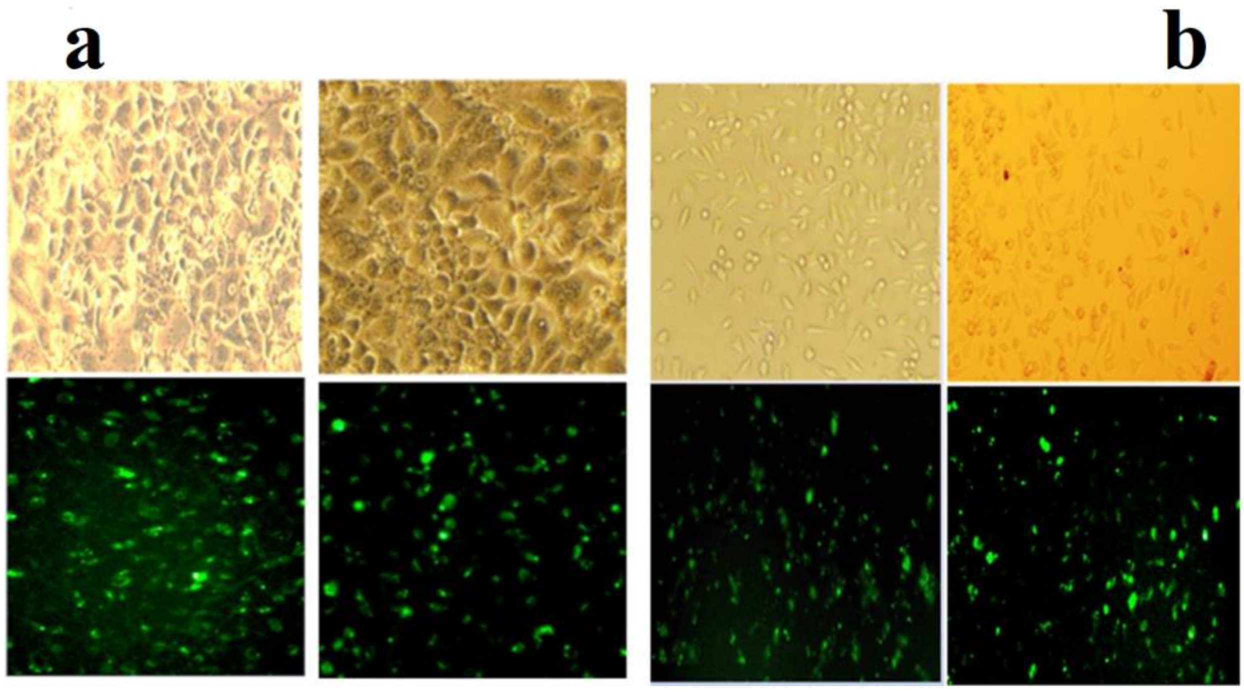

Supplement: Supplementary file 1 — Additional file 1: Figure S1. MCF-7 & MDA-MB-231 cell lines transfected with FAM-labeled microRNAs. Fluorescence microscopy images of MCF-7 and MDA-MB-231 cells transfected with miR-154 mimic and miR inhibitor negative controls (NC) labeled with FAM. a) Evaluation of transfection efficiency under fluorescence and light microscope in MCF-7 cells transfected with FAM-labeled miR-154 mimic NC and miR-inhibitor NC. b) Evaluation of transfection efficiency, under fluorescence and light microscope, in MDA-MB-231 cells transfected with FAM-labeled miR-154 mimic NC and miR-inhibitor NC. The fluorescence microscopy images were obtained 24 h after transfection. Table S1. primer sequences for real-time PCR, and primer sequences used for amplification and cloning of NAMPT 3′-UTR. The underlined sequences represent the restriction enzyme recognition sites (CTCGAG for XhoI and GCGGCCGC for NotI) added for cloning into psiCHECK-2. [file 12885_2019_6221_MOESM1_ESM.pdf]
